# Genetic Diversity of Whiteflies Colonizing Crops and Their Associated Endosymbionts in Three Agroecological Zones of Cameroon

**DOI:** 10.3390/insects15090657

**Published:** 2024-08-30

**Authors:** Lanvin R. K. Kepngop, Everlyne N. Wosula, Massoud Amour, Pierre G. T. Ghomsi, Louise N. Wakam, Germain Kansci, James P. Legg

**Affiliations:** 1 Laboratory for Phytobiochemistry and Medicinal Plants Studies, Antimicrobial & Biocontrol Agents Unit (AmBcAU), Department of Biochemistry, Faculty of Science, University of Yaoundé 1, Yaoundé P.O. Box 337, Cameroon; 2 International Institute of Tropical Agriculture, Dar es Salaam P.O. Box 34441, Tanzania; 3 Laboratory of Food Science and Nutrition, Department of Biochemistry, Faculty of Science, University of Yaoundé 1, Yaoundé P.O. Box 337, Cameroon

**Keywords:** *Bemisia tabaci*, KASP, *Arsenophonus*, *Wolbachia*, *Rickettsia*

## Abstract

**Simple Summary:**

*Bemisia tabaci* is as a major pest of vegetable crops in Cameroon, and several species have developed resistance against insecticides. Here, we investigated the frequency of infection by endosymbiont and the genetic diversity of whiteflies in Cameroon. Mitochondrial cytochrome oxidase I (mtCOI) markers and Kompetitive Allele Specific PCR (KASP) were used for the characterization. Overall, an analysis of the mtCOI sequences showed six mitotypes of *Bemisia tabaci*, and two distinct clades of *Bemisia afer* and *Trialeurodes vaporariorum*. *Bemisia tabaci* mitotypes identified included: Mediterranean (MED) on tomato, pepper, okra, and melon; and sub-Saharan Africa (SSA) groups and sub-groups (SG)–SSA1-SG1, SSA1-SG2, SSA1-SG5, SSA3, and SSA4 on cassava. The six mitotypes of cassava *B. tabaci* were split into three SNP haplogroups including sub-Saharan Africa–West Africa (SSA-WA), sub-Saharan East and Central Africa (SSA-ECA), and SSA4 by KASP genotyping. The endosymbionts identified infecting the whiteflies were *Arsenophonus, Rickettsia*, and *Wolbachia*.

**Abstract:**

*Bemisia tabaci* (Gennadius) is as a major pest of vegetable crops in Cameroon. These sap-sucking insects are the main vector of many viruses infecting plants, and several cryptic species have developed resistance against insecticides. Nevertheless, there is very little information about whitefly species on vegetable crops and the endosymbionts that infect them in Cameroon. Here, we investigated the genetic diversity of whiteflies and their frequency of infection by endosymbionts in Cameroon. Ninety-two whitefly samples were collected and characterized using mitochondrial cytochrome oxidase I (mtCOI) markers and Kompetitive Allele Specific PCR (KASP). The analysis of mtCOI sequences of whiteflies indicated the presence of six cryptic species (mitotypes) of *Bemisia tabaci*, and two distinct clades of *Bemisia afer* and *Trialeurodes vaporariorum*. *Bemisia tabaci* mitotypes identified included: MED on tomato, pepper, okra, and melon; and SSA1-SG1, SSA1-SG2, SSA1-SG5, SSA3, and SSA4 on cassava. The MED mitotype predominated in all regions on the solanaceous crops, suggesting that MED is probably the main phytovirus vector in Cameroonian vegetable cropping systems. The more diverse cassava-colonizing *B. tabaci* were split into three haplogroups (SNP-based grouping) including SSA-WA, SSA4, and SSA-ECA using KASP genotyping. This is the first time that SSA-ECA has been reported in Cameroon. This haplogroup is predominant in regions currently affected by the severe cassava mosaic virus disease (CMD) and cassava brown streak virus disease (CBSD) pandemics. Three endosymbionts including *Arsenophonus, Rickettsia*, and *Wolbachia* were present in female whiteflies tested in this study with varying frequency. *Arsenophonus*, which has been shown to influence the adaptability of whiteflies, was more frequent in the MED mitotype (75%). *Cardinium* and *Hamiltonella* were absent in all whitefly samples. These findings add to the knowledge on the diversity of whiteflies and their associated endosymbionts, which, when combined, influence virus epidemics and responses to whitefly control measures, especially insecticides.

## 1. Introduction

The whitefly *Bemisia tabaci* (Gennadius) (Hemiptera: Aleyrodidae) is a significant pest that damages many plants worldwide such as ornamentals, vegetables, legumes, and cotton [1]. Whitefly species of this complex are extremely polyphagous insects, and they are found in greenhouses and crop fields in temperate and tropical regions [2,3]. In sub-tropical and tropical countries, *B. tabaci* is one of the principal pests, especially on the main food and cash crops such as cassava, cotton, sweet potato, tobacco, and tomato [4]. These whiteflies have a severe impact on the economic activity and food security of many farmers and populations, since agriculture is one of the main economic activities [4]. *Bemisia tabaci* whiteflies have the ability to carry viruses with semi-persistent and persistent mechanisms and can vector viruses belonging to at least five genera (*Begomovirus*, *Crinivirus*, *Ipomovirus*, *Carlavirus*, and *Torradovirus*) [5]. Their sap-sucking feeding also causes physical damage to plants, although the most damage caused is by the vectoring of over 300 plant viruses [1]. Economic losses due to *B. tabaci* were estimated at USD 10 billion from 1980 to 2000 [6] in the US, and over USD 1 billion annually on cassava in Africa [7]. *Bemisia tabaci* has been described as a cryptic species complex with more than 39 morphologically indistinguishable species [3,8,9]. Most of these species are localized in geographic regions **[10]** except a few, among which two occur worldwide and are invasive: the “Mediterranean” or “MED” species (previously referred as ‘Biotype Q’) and the “Middle East–Asia Minor” species (“MEAM1”—previously referred to as ‘Biotype B’) [2]. Among these whitefly species, there are many that colonize Solanaceae [11,12] and these have shown the ability to rapidly develop resistance to chemical insecticides [4,13]. In Africa, despite the harmful impact on potential natural enemies and on the environment, chemical pesticides are widely used to control *B. tabaci* populations. Whiteflies are infected with facultative endosymbiont bacteria that have been implicated in their pest status, as they have been shown to affect tolerance to insecticides [14], virus transmission efficiency [15,16], and high-temperature tolerance [17]. As with many other arthropods, endosymbiotic bacteria are also widespread in *B. tabaci* [18]. Endosymbionts are present in many parts of insects, such as the whitefly body cavity, hemolymph, or intracellularly in special cells called bacteriocytes [19]. Members of the *Bemisia* species complex carry a primary endosymbiotic bacterium called *Candidatus Portiera aleyrodidarum* [20,21], which is fixed in populations and confined to bacteriocyte cells, with a main role in the regulation of amino acid-deficient diets in all whiteflies [20]. This bacterium is essential for host survival and development and has a long co-evolutionary history with all members of the subfamily Aleyrodidae [20,22,23]. In addition to the primary endosymbiont, many different secondary endosymbionts may be present, such as *Rickettsia* [24], *Wolbachia* [25,26], *Hamiltonella* and *Arsenophonus* [20,21], *Cardinium* [27], and *Fritschea* [28]. These have been reported from *Bemisia* populations around the world. Several of these secondary endosymbionts interfere with host physiology, ecology, and reproduction [29,30,31], and they may have effects on rapid evolutionary shifts [32], thermotolerance [17], resistance to insecticides [14], host fitness [33], defense against pathogens [34], and virus transmission ability [15,35,36,37].

Several *Bemisia* mitotype populations around the world have been surveyed for infection with endosymbionts and showed a clear variation in the infection frequency within the *Bemisia* genetic groups [18,38,39,40]. For example, in populations tested from China, *Wolbachia*, *Rickettsia*, *Arsenophonus*, *Hamiltonella*, and *Cardinium* were detected in MEAM1 and MED populations [41]. *Arsenophonus*, *Cardinium*, *Rickettsia*, and *Wolbachia* were detected in native whiteflies of Africa [39], China [38], and India [42], but not *Hamiltonella* and *Fritschea*. A study by Gorsane et al. [43] hypothesized that the presence of *Cardinium* in MEAM1 and *Cardinium*, *Fritschea*, and *Wolbachia* in MED may explain the differences in infestation status possibly due to plant host variability, site to site variations, and the influence of chemical insecticides. Also, *Hamiltonella* in MED and *Rickettsia* in MEAM1 populations are also reported to increase the acquisition, retention, and transmission of the Tomato yellow leaf curl virus [44,45]. However, recent work on cassava whiteflies indicated that the coinfection of *Bemisia tabaci* colonies of sub-Saharan Africa 1 sub-group 3 (SSA1-SG3) by two secondary endosymbiotic bacteria *Arsenophonus* and *Rickettsia* reduced their ability to transmit East African cassava mosaic virus—Uganda (EACMV-UG) and these whiteflies also showed lower adult emergence, slower development, and lower virus retention abilities than those free of bacteria [46]. Skaljac et al. [47] showed that *Hamiltonella* and *Arsenophonus* were strictly localized to the bacteriocytes during all developmental stages in *T. vaporariorum.* However, they are less likely to be able to manipulate their host’s reproduction since this requires invading reproductive organs outside the bacteriocyte. This observation suggests that *Hamiltonella* and *Arsenophonus* in *T. vaporariorum* are involved in a functional advantage rather than its reproduction [47].

For effective whitefly management, therefore, it is important to make the correct species identification, including the identification of intracellular bacterial communities. Many aspects of this species complex remain unknown, such as the degree of genetic isolation between some species, their geographical distribution, as well as the within-species genetic diversity and endosymbiont species being carried by whiteflies. Knowing which species of whitefly is present in Cameroon and its associated endosymbionts is crucial for the management of this pest. Because these cryptic species of whitefly are morphologically indistinguishable [3], we used molecular tools (mitochondrial cytochrome oxidase I sequences—mtCOI and Kompetitive Allele Specific PCR–KASP) for identification. In the study reported here, the endosymbiont colonization frequency and results from whitefly mtCOI sequencing and KASP were combined to provide an overall assessment of the genetic diversity of *B. tabaci* in Cameroon.

## 2. Materials and Methods

### 2.1. Whitefly Sampling and Population Study

#### 2.1.1. Field Survey

Field surveys were conducted in four regions of Cameroon by using standardized diagnostic protocols described by many authors [48,49,50]. This involved collecting data and sampling (counting whiteflies and collecting specimens for molecular identification) from cassava, tomatoes, pepper, and okra young plants. The number and distribution of collection sites varied, according to the number of fields that were found in each location and the relative abundance of *B. tabaci* in those fields. A total of 24 fields were randomly chosen and surveyed from the four regions. Whitefly samples were collected from fifteen fields of cassava (*Manihot esculenta* Crantz), four fields of tomato (4) (*Solanum lycopersicum* L.), three fields of okra (*Abelmoschus esculentus* L.), one field of melon (*Cucumis melo* L.) and one field of pepper (*Capsicum annuum* L.) across twelve locations in three (monomodal rainforest, bimodal rainforest, and highland) agroecological zones across Cameroon (Table 1). The locations included Ngoa Ekelle, Minkoameyos, Awae, Bangangte, Manko’o, Mantem, Nyang, Ngohsi, Djibeeng, Balngong, Bandoumou, and Melen. *Bemisia tabaci*-colonizing tomatoes were collected from two locations and four fields (Manko’o in the highland agroecological zone and Awae in the bimodal rainforest agroecological zone). *Bemisia tabaci* on cassava were sampled from 10 locations and 15 fields (Ngoa Ekelle, Minkoameyos, Awae, Balngong, and Bandoumou, all located in the bimodal rainforest agroecological zone, Bangangte in the highland agroecological zone, and Mantem, Nyang, Ngohsi, and Djibeeng in the monomodal rainforest). On okra, *B. tabaci* were collected from two locations and three fields (Minkoameyos and Awae located in the bimodal rainforest agroecological zone). On melon, *B. tabaci* were collected from one location and one field (Ngoa Ekelle in the bimodal rainforest agroecological zone). On pepper, *B. tabaci* were collected from one location and one field (Melen in the bimodal rainforest agroecological zone).

#### 2.1.2. Data Recording and Storage

In each field, 20 plants were randomly selected along two diagonal transects across the field and whiteflies were counted from the top five leaves of vegetable and for cassava. Approximately 40 *B. tabaci* adult whiteflies were collected from each field and all whiteflies collected from a single field were considered to be a single sample. Whiteflies were aspirated alive and immediately preserved in 95% ethanol in vials, before being stored in the freezer at −20 °C.

### 2.2. Genetic Diversity of Sampled Whiteflies

#### 2.2.1. DNA Extraction

DNA extraction was carried out in the molecular laboratory at the International Institute of Tropical Agriculture (IITA) in Dar es Salaam, Tanzania. The insects (single female whiteflies) were added to 3 µL of lysis buffer in a 1.5 mL Eppendorf tube, then macerated and another 20 µL of lysis buffer was added. The lysis buffer contained 10 mM Tris-HCl (pH 8.0, 50 mM KCL, 2.5 mM MgCl, 0.45% Tween–20, 0.01% Gelatine, and 60 µg/mL Proteinase). The mixture was then vortex shaken and spun down and immediately incubated on ice for 15 min. This was followed by incubation at 55 °C in a water bath for 30 min. The lysate was then stored at −20 °C for downstream use. For PCR use, the lysate was diluted while using sterile diethylpyrocarbonate (DPEC) treated water in a ratio of 1:9.

#### 2.2.2. MtCOI Amplification and Sequencing

PCR products of the mtCOI fragment were produced using the forward primer 2195-Bt-F (5′-TGRTTTTTTGGTCATCCRGAAGT-3′) and C012-Bt-sh2-R (5′-TTTACTGCACTTTCTGCC-3′) [51] to target *Bemisia* whiteflies (~850 bp), and universal primers LCO-1490-F (5′-GGTCAACAAATCATAAAGATATTGG-3′) and HCO-2198-R (5′-TAAACTTCAGGGTGACCAAAAAATCA-3′) to target non-*Bemisia* whiteflies (~710 bp) using a thermocycler (Applied Biosystems™ GeneAmp^R^ PCR system 9700, Foster City, CA, USA), under the following conditions: first cycle of denaturation at 95 °C for 5 min, followed by 35 cycles of denaturation at 94 °C for 40 s, and annealing at 54 °C for 30 s, 72 °C for 45 s, and the final extension at 72 °C for 10 min. A total reaction mixture of 25 µL was made up of 1X QuickLoad Master Mix (New England Biolabs, Hitchin, UK), 1 mM MgCl, 0.24 µM of each primer, 2 µL DNA, and nuclease-free water.

The PCR products were electrophoresed in a 1% agarose gel stained in GelRed (Biotium, Hayward, CA, USA) at 100 V for 30 min in gels buffered with a 1 × TAE buffer. DNA bands were visualized under ultraviolet light (UVP GelStudio PLUS, Analytik Jena, Upland, CA, USA) and only samples with intact bands were selected for sequencing. PCR products were sent to Psomagen Inc. (Rockville, MD, USA) for purification and direct sequencing. DNA sequences were manually edited using Ridom Trace Edit v1.1.0 software (Ridom GmbH., Würzburg, Germany). The sequences were assembled into contigs using CLC Main Workbench 22 (QIAGEN, Aarhus, Denmark). A multiple alignment of the edited sequences was performed using Clustal W in MEGA version 7 [52] and the sequences were trimmed. The construction of a maximum-likelihood phylogenetic tree was performed using MEGA with 1000 bootstrap replicates. Sequences were blasted using GenBank’s (NCBI) Blastn and selected reference sequences with 99% to 100% identity to our mtCOI sequences were included in the phylogenetic tree for comparison with previously published haplotypes. The outgroup *Bemisia afer* was included in the phylogenetic tree for *Bemisia tabaci*, while the outgroups *Bemisia afer*, *B. tabaci*, and *Aleurodicus dispersus* were included for *Trialeurodes vaporariorum*.

#### 2.2.3. Kompetitive Allele-Specific PCR (KASP)

Kompetitive Allele-Specific PCR (KASP) was used to further distinguish the major genotypes of cassava-colonizing *B. tabaci* [53]. The KASP reaction mixture (10 μL) contained 5 μL 2X KASP master mix, 0.14 μL KASP primer assay mix, and 5 μL DNA template (1 μL of PCR product/DNA extract + 4 μL of sterile water). KASP genotyping was performed in a Strategene MX 3000P real-time PCR unit (Agilent Technologies, Santa Clara, CA, USA). The following cycling conditions were used: Stage 1: 30 °C for 60 s (pre-read); Stage 2: 94 °C for 15 min hot-start Taq activation (1 cycle); Stage 3: 94 °C for 20 s, 61 °C (61 °C decreasing 0.6 °C per cycle to achieve a final annealing/extension temperature of 55 °C) for 60 s (10 cycles); Stage 4: 94 °C for 20 s, 55 °C for 60 s (29 cycles); Stage 5: 94 °C for 20 s, 57 °C for 60 s (3 cycles); and Stage 6: 37 °C for 60 s (1 cycle, cooling) followed by an end-point fluorescent read. These conditions were used for three primers (BTS99-319, BTS22-762, and BTS55-473), while Stage 3: 94 °C for 20 s, 68 °C (68 °C decreasing 0.6 °C per cycle to achieve a final annealing/extension temperature of 62 °C) was used for the primer BTS613. The quality of genotyping cluster plots was visually assessed, and only samples in distinct clusters were considered for manual SNP calling, using the MxPro -Mx3000P software incorporated in the Strategene MX 3000P unit (Agilent Technologies, Santa Clara, CA, USA) and KlusterCaller (LGC Genomics, Teddington, UK). The KASP protocol for *B. tabaci* is described in detail in Wosula et al. [53].

### 2.3. Infectivity of Endosymbionts in Whiteflies

#### Screening for the Presence of Endosymbionts by PCR

The DNA extracts from 75 whitefly specimens that were identified through mtCOI sequencing were used for bacterial endosymbiont diagnoses. The PCR was performed using a total volume of 25 μL containing 2 μL template DNA, 12.5 μL OneTaq Quick-Load 2X Master Mix (New England Biolabs, Hitchin, UK) with Standard Buffer, 0.6 μL of primer (0.25 mM) (Table 2), 1 μL MgCl2 (25 mM) solution, and 8.9 μL of sterile water. A total of 35 cycles of amplification were carried out in a Veriti 96-Well Thermal Cycler (Applied Biosystems, Foster City, CA, USA), and conditions were the same for all sets of primers except for the annealing temperature (Table 2): denaturation at 95 °C for 3 min and 94 °C for 30 s, annealing temperature as showed in Table 2 for 45 s, and extension at 72 °C for 1 min, and a final extension at 72 °C for 7 min and held at 10 °C.

Amplified PCR products were separated using 1% agarose gel electrophoresis, stained with GelRed (Biotium, Hayward, CA, USA) with a 100 bp ladder (NEB, England, UK), and then visualized under ultraviolet light (UVP GelStudio PLUS, Analytik Jena, Upland, CA, USA). All PCRs included a negative control (sterile water) to spot any DNA contamination, and a positive control to prevent false negatives.

## 3. Results

### 3.1. Whitefly Abundance

Mean whitefly counts varied with survey site (Table 3). The mean whitefly count per plant across the country was 8.0 for cassava, 29.7 for tomato, 31.6 for okra, 60.0 for melon, and 18.0 for pepper. At the regional level, the mean whitefly count varied from 2.9 in the Littoral region to 12.5 in the southwest region for cassava. In addition, mean whitefly counts on vegetable crops such as tomato, pepper, melon, and okra were high and ranged from 18 to 60. The highest whitefly mean count of 60 was recorded for the center region on Melon, while the Littoral region had the lowest mean (2.9) except on cassava. Field-level data showed many fields with whitefly counts higher than 50 per plant located in the center, southwest, and west regions.

### 3.2. mtCOI Mitotypes of Whiteflies Colonizing Five Crop Plants in Cameroon

**Figure 1:**
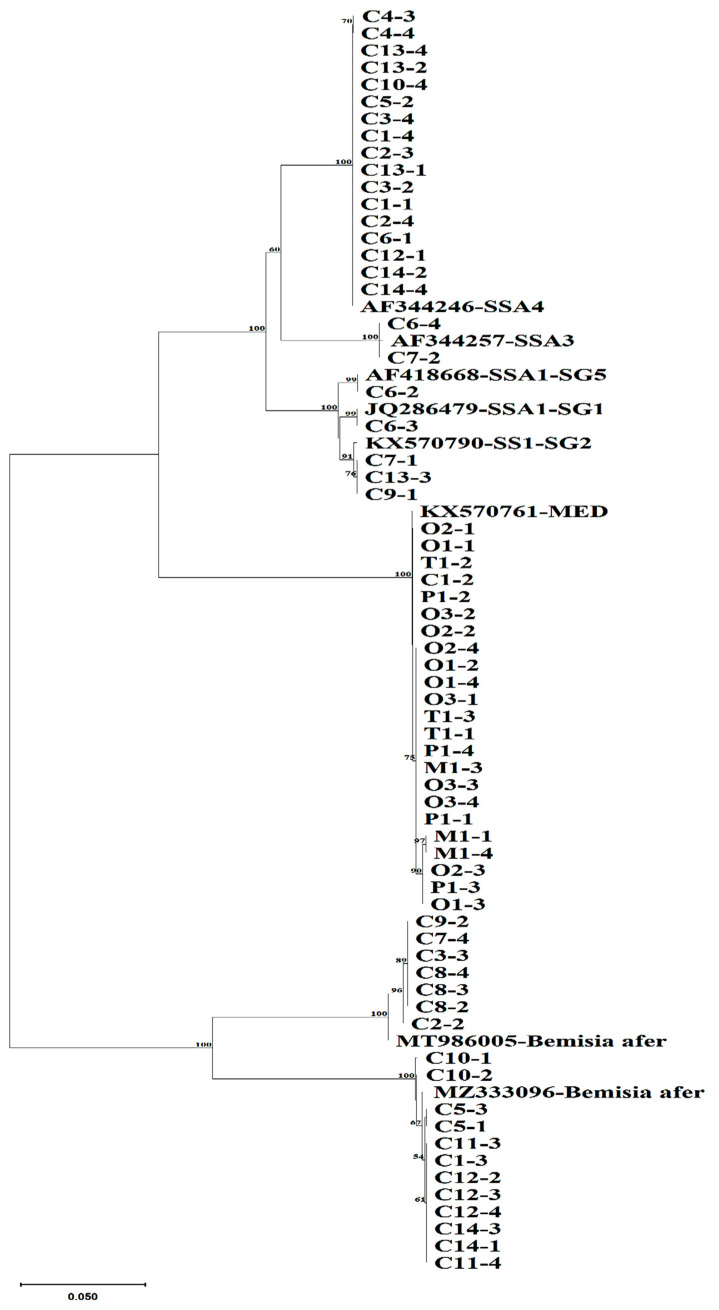
Phylogenetic relationships of the *Bemisia tabaci* whiteflies collected from crop plants in three agroecological zones of Cameroon. C (cassava plant), O (okra plant), T (tomato plant), P (pepper), and M (melon). The first number represents the field, and the second number is the sample number.

**Figure 2:**
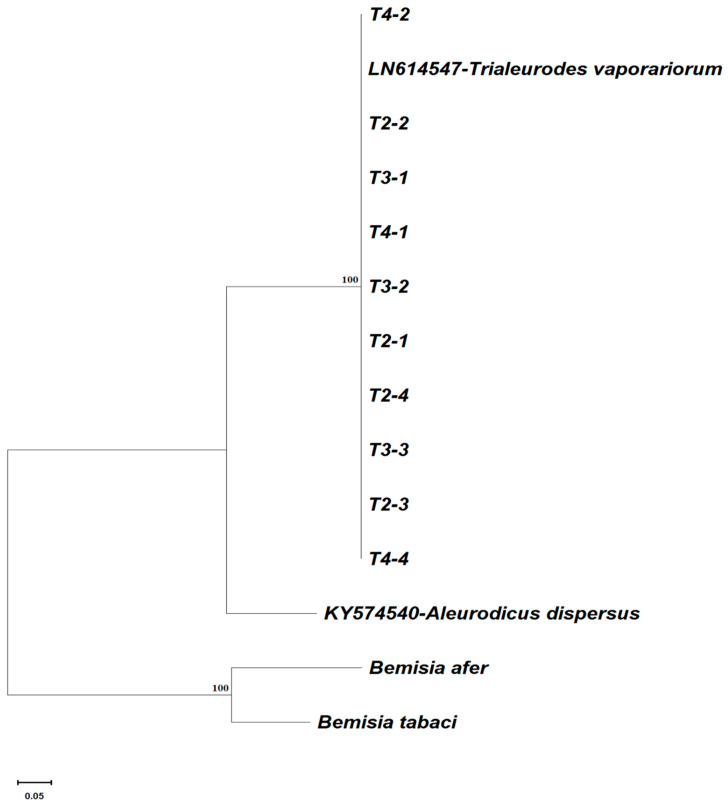
Phylogenetic relationships of the *Trialeurodes vaporariorum* whiteflies collected from tomato plants in three agroecological zones of Cameroon.

**Figure 3:**
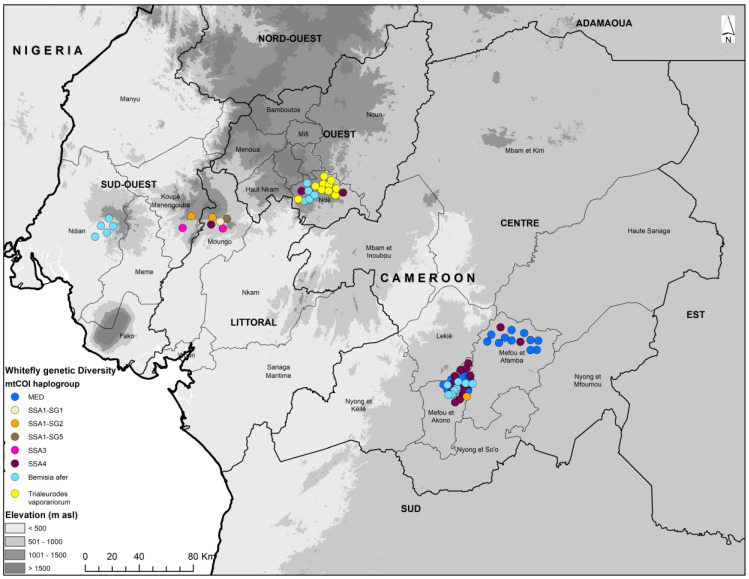
Genetic diversity according to mtCOI sequences of whiteflies collected on cassava, okra, tomato, pepper, and melon in Cameroon.

In total, 92 whitefly samples were sequenced, out of which 75 produced quality mtCOI sequences. There was a high level of diversity among *B. tabaci* populations that were collected from the sampled crop plants. The sequences obtained from whiteflies collected from pepper, okra, and melon were grouped into one phylogenetic group (MED), but whiteflies from tomato were grouped into two phylogenetically distinct groups: *B. tabaci* MED and *Trialeurodes vaporariorum* (Westwood). The sequences from cassava were grouped into five mitotypes of *B. tabaci* (SSA1-SG1, SSA1-SG2, SSA1-SG5, SSA3, and SSA4) and two *Bemisia afer* (Priesner and Hosny) clades (Figure 1, Figure 2 and Figure 3). These groups were identified based on the topology of the phylogenetic tree and the clustering of the sequences that were obtained from this study relative to the reference sequences retrieved from GenBank. The predominant *B. tabaci* mitotype MED had a total of 23 whiteflies, which accounted for 30.3% of all the whiteflies collected from the four host plants (tomato, okra, melon, and pepper) and they were distributed in the two agroecological zones where these samples were collected (bimodal rainforest and highland). The second most abundant *B. tabaci* mitotype was SSA4 with 17 whiteflies (22.4%), with all of them being found on cassava, and they were distributed in all three agroecological zones (monomodal rainforest, bimodal rainforest, and highland). The other mitotypes occurring on cassava were SSA1-SG1 (1.3%) (monomodal rainforest agroecological zone), SSA1-SG2 (4.0%) (monomodal rainforest and bimodal rainforest agroecological zone), SSA1-SG5 (1.3%) (monomodal rainforest agroecological zone), and SSA3 (2.6%) (monomodal rainforest agroecological zone). *Bemisia afer* had two distinct clades with 19 whiteflies, which accounted for 25.3% of the sequences. It was present on cassava in all three sampled regions (monomodal rainforest, bimodal rainforest, and highland agroecological zone). *Trialeurodes vaporariorum* (10 whiteflies accounting for 13.2% of the sequences) was present only on tomato in the highland agroecological zone. Sequences from this study were deposited in the NCBI database accessions PP580858–PP580933.

### 3.3. Kompetitive Allele Specific PCR Analysis (KASP)

**Figure 4:**
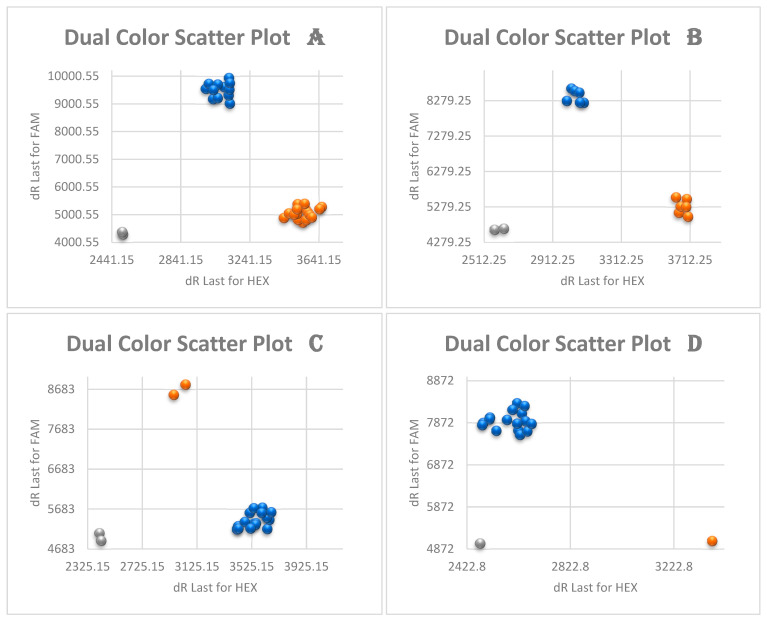
Cluster plots of four KASP SNP-based primers discriminating between major haplogroups of *Bemisia tabaci* whiteflies collected from cassava. Orange and blue represent the two distinct alleles, while grey dots represent negative controls. (**A**) = BTS99-319 (SSA-ECA and SSA-WA vs. SSA-ESA, SSA-CA, SSA2, and SSA4); (**B**) = BTS22-762 (SSA-ECA vs. SSA-WA); (**C**) = BTS613 (SSA-ESA and SSA-CA vs. SSA-ECA, SSA-WA, SSA2, and SSA4); and (**D**) = BTS55-473 (SSA2 vs. SSA4).

**Figure 5:**
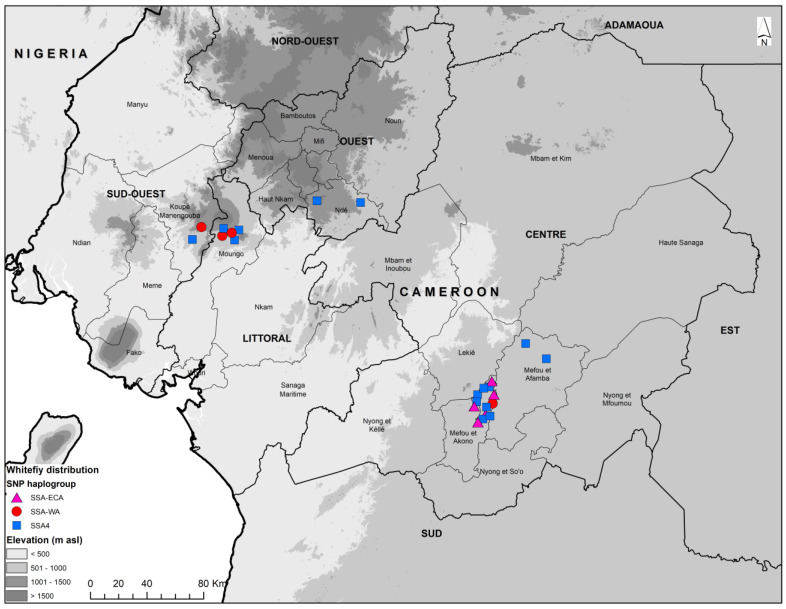
Cassava whiteflies (*Bemisia tabaci*) haplogroup distribution based on KASP SNP genotyping discrimination.

The KASP genotyping results are based on SNP genotyping, and it has been designed to discriminate between the major genotypes of cassava *B. tabaci* whitefly. Twenty-four whiteflies that were identified as cassava *B. tabaci* based on mtCOI sequencing were further characterized using KASP genotyping. The SNP genotyping clusters for the selected four primers for representative samples are presented in Figure 4 and Figure 5.

KASP genotyping split the 24 cassava whitefly samples into three haplogroups: SSA-ECA, SSA-WA, and SSA4. Haplogroup SSA4 with 15 samples out of the 24 (62.5%) was the most frequent, and included mtCOI mitotypes SSA4 (10), SSA3 (2), SSA1-SG2 (2), and SSA1-SG5 (1). The haplogroup SSA-ECA was the second most frequent with five samples (20.8%), all of which were designated as mitotype SSA4. The last haplogroup SSA-WA had four samples (16.7%) that were designated as mitotypes SSA4 (2), SSA1-SG2 (1), and SSA1-SG5 (1). This is the first study to report the designation of mitotype SSA4 samples into SNP haplogroup SSA-ECA (Table 4).

The samples in the SSA4 group were from Ngoa Ekelle, Minkoameyos, Awae, Bangangte, Mantem, Nyang, Djibeeng, and Bandoumou located in the three agroecological zones. Samples in the SSA-ECA group were from Ngoa Ekelle, Balngong, and Bandoumou in the central part of Cameroon located in the bimodal rainforest agroecological zone, while those in SSA-WA were from Mantem and Nyang in the monomodal rainforest agroecological zone of western Cameroon, as well as from Ngoa ekelle and Awae in the bimodal rainforest agroecological zone in the central part of the country.

### 3.4. Frequency of Infection of Whiteflies by Three Endosymbionts

**Figure 6:**
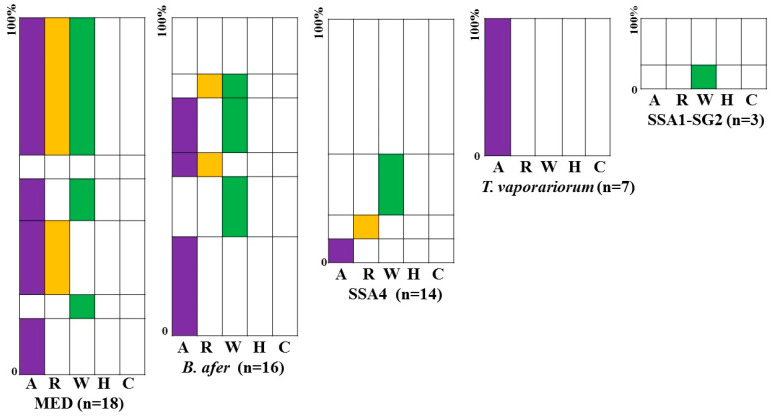
The frequency of infections of symbionts in different whitefly populations. The number of individuals tested and the genetic groups they belong to are indicated below each graph. The various colors represent the different secondary symbionts tested where A, *Arsenophonus*; R, *Rickettsia*; W, *Wolbachia*; H, *Hamiltonella*; and C, *Cardinium*. The combination of colors in the rows represents whiteflies that shared that particular complex of symbionts. The width of the row indicates the percentage of individuals that have that combination of symbionts. The numbers in brackets indicate the number of individuals tested in each of the genetic groups and sub-groups.

The primary endosymbiont *Portiera* was detected in 77% of the whiteflies identified by mtCOI and KASP (75). Therefore, only whiteflies with at least three specimens and bearing *Portiera* were used to evaluate the presence of secondary endosymbionts. The secondary endosymbionts were found in 78% (45 whiteflies infected out of 58 tested) of the insects, and their frequency varied significantly across the different whitefly populations. The SSA4 mitotype showed the highest percentage of no secondary endosymbiont with 50% of non-infection. However, none of the whitefly samples showed an infection by the endosymbionts *Hamiltonella* and *Cardinium* (Figure 6). The identified endosymbionts were more often in single infections in cassava-colonizing whiteflies than non-cassava whiteflies, except in *T. vaporariorium* where *Arsenophonus* singly infected 100% (7/7) of the specimens. In the MED mitotype, infection by the combination of *Arsenophonus* (A), *Wolbachia* (W), and *Rickettsia* (R) was the most represented (41%). Only A (18%) and W (6%) were identified in single infection and all other infections were in coinfection with *Arsenophonus* AR (23%) and AW (12%). Whiteflies collected on cassava and identified as *B. afer* had infections with all of the endosymbionts dominated by the single infections of A (38%) and W (23%). No triple infection was recorded but all possible double infections were detected as AR (8%), AW (23%), and RW (8%). The SSA4 mitotype was singly infected with *Wolbachia* (W) in 72% of the specimens followed by *Arsenophonus* (A) and *Rickettsia* (R) with 14% each. The SSA1-SG2 mitotype had single infections of W (33%) but 67% of this mitotype were not infected by the secondary endosymbiont (Figure 6). Considering only infected whiteflies, *Arsenophonus* (95.8%; 23/24) was the most frequently represented endosymbiont in non-cassava whiteflies (MED, *T. vaporariorium*). However, in cassava whiteflies (SSA4, SSA1-SG2, and *Bemisia afer*) *Wolbachia* was the most frequently represented (56.5%; 13/23).

## 4. Discussion

The current study confirmed that whiteflies occur widely on vegetables and cassava throughout Cameroon, and their abundance depends on the host plant species and agroecological region. The number of whiteflies was higher on vegetables compared to cassava; this could be attributed to the fact that the mitotype MED found on vegetables is an invasive species known for rapid resistance development to insecticides, hence high populations [3]. The abundance of whiteflies in vegetables is linked with increased spread and the severity of virus diseases [59,60].

An analysis of the genetic diversity of the whiteflies collected showed that the three whitefly species commonly occurring on cassava and vegetables were *B. tabaci*, *B. afer*, and *T. vaporariorum*. Whilst *B. afer* and *B. tabaci* occurred widely throughout the agroecological zones sampled, *T. vaporariorum* was only reported from tomato in the highland zone. *T. vaporariorum* is commonly called the greenhouse whitefly and has a more temperate distribution than the two *Bemisia* species. In Africa, *T. vaporariorum* only occurs in high altitude and cooler regions [61].

*Bemisia afer* accounted for 25% of the *Bemisia* spp in samples from Cameroon and it was identified in all of the agroecological zones. Although *B. afer* is not currently considered to be a significant threat to cassava production in Africa, it has been shown to be an economically important viral vector in other crops, transmitting the sweet potato chlorotic stunt virus in sweet potatoes in Peru [62]. This highlights the importance of careful monitoring of this second cassava-colonizing *Bemisia* species in Africa.

The clustering of the cassava *B. tabaci* whitefly mitotypes SSA1-SG1, SSA1-SG2, SSA1-SG5, SSA3, and SSA4 into a distinct major clade separate from *B. tabaci* whiteflies that do not colonize cassava is consistent with what has been reported in other studies of *B. tabaci* from various cassava-growing countries in Africa [57,63]. The grouping of the MED mitotype is also consistent with what has been reported in previous studies [63]. We found that *B. tabaci* MED was predominant on okra, pepper, tomato, and melon in all of the sampled locations. MED is a globally important *B. tabaci* mitotype, which is thought to have originated from countries neighboring the Mediterranean basin, which include Algeria, Morocco, Egypt, and Sudan in Africa [3,64]. Consequently, there are numerous other reports of its prevalence on a wide range of crop and weed hosts [55,63,65,66,67]. *B. tabaci* MED has been reported to be extremely polyphagous and invasive [3], causing damage to both field and greenhouse crops [68]. It has also developed resistance to various insecticides under intensive production systems [69,70,71]. The mitotype *B. tabaci* MED was also the most abundant whitefly collected. As confirmed by the results on whitefly abundance, evidence elsewhere has suggested that begomovirus infection can increase *B tabaci* MED fecundity, which facilitates its spread [72]. The predominance of MED on major vegetable crops considered in this study confirms that this is the most important whitefly pest of vegetables in Cameroon.

In the studied cassava group of *B. tabaci*, the largest number of samples based on mtCOI sequencing were in the SSA4 mitotype, and these were widely distributed across the sampled locations. SSA4 has previously been reported on cassava in Cameroon accounting for 15% [73] and 37% [56] of the cassava whitefly samples. The findings from this study suggest SSA4 is increasing in dominance compared to other mitotypes. SSA1-SG1 and SSA1-SG5 were less frequent, as they were only detected at a single location each. In another study, a similar trend was reported for samples obtained from cassava in Cameroon, where 7% were identified as SSA1-SG1, while none were identified as SSA1-SG5 [56]. These results differ from other recent findings from East and Central Africa, which have shown SSA1-SG1 to be the predominant *B. tabaci* mitotype on cassava [7,54,57,74]. SSA2 was not encountered in the current study, although this may be a consequence of the absence of samples from northern parts of Cameroon. A similar result was reported elsewhere [73], where only SSA3 and SSA4 were recorded, although other research on cassava *B. tabaci* in Cameroon, which included samples from the north of the country, recorded SSA2 making up 44% of the total types found [56]. This trend of presence or absence of SSA2 depending on the duration of the surveys is not unique; it has been observed in East Africa where SSA2 was reported as being absent in samples collected from cassava [75,76], and then reported present with subsequent surveys [51,56,57,77]. In South Sudan, SSA2 was reported as the most predominant mitotype on cassava accounting for 75% of the samples that were collected from the cassava plants [55]. Similarly, the current study noted the presence of SSA1-SG5 and SSA1-SG2, which were not found in previous studies [56,57], although, importantly, the sample collection locations differed between the studies. An accurate identification of these species is critical for the effective management of whiteflies both as pests and as virus vectors. The development of the KASP diagnostic method, based on a large SNP dataset for *B. tabaci* in Africa, provided important means for distinguishing between the major genetic groupings of *B. tabaci* occurring on cassava in Africa [53]. This method allows for the identification of these haplogroups in laboratory procedures lasting a matter of hours and with no requirement for sequencing. This study builds on the importance of adopting KASP as a diagnostic method for cassava *B. tabaci* whiteflies as it reports for the first time mitotype SSA4 samples designated as SNP haplogroups SSA-ECA and SSA-WA (Table 4). An important outcome of SNPs’ analysis and the application of KASP has been the recognition of an association between the haplogroup SSA-ECA and regions currently affected by severe CMD and CBSD pandemics [56].

The most common SNP haplogroup, however, was SSA4. For the first time, this haplogroup included samples that were designated as mitotypes SSA1-SG5 and SSA1-SG2. Previously, samples identified as SSA4 using KASP, based on SNPs’ analysis, only had mitotypes SSA3 and SSA4 [53,56]. There were similar novel associations between SSA-WA and mitotype SSA4; previously, SSA-WA was reported to have mitotypes SSA1-SG5 (predominantly), SSA1-SG1, and SSA2 [56]. The present study also revealed that haplogroup SSA-ECA had all samples designated as mitotype SSA4. This is a first, as previously this SNP haplogroup only had samples of mitotypes SSA1-SG1, SSA1-SG2, and SSA1-SG1/SG2 [54,55,56]. Each of these sets of results provides further evidence of the weak association between identifications based on SNPs dispersed throughout the *B. tabaci* genome and identities derived from short, maternally-inherited mitochondrial COI sequences. Finally, this work shows, again, that there is not a good correlation between COI and KASP identities, which confirms the unreliability of using COI to identify *B. tabaci* genotypes.

The identification of *B. tabaci* haplogroup SSA-ECA raises a concern about the potential future spread of cassava viruses in Cameroon, as SSA-ECA is predominant in areas associated with severe epidemics of CMD and CBSD in East, Central, and Southern Africa [54,56]. It is important to note, however, that CBSD has not yet been identified in Cameroon and has only so far been reported from East and Southern Africa. The most westerly report of CBSD has been made from the eastern part of the Democratic Republic of Congo (DRC) [78].

Microbial endosymbionts represent an important component of the biology and ecology of invertebrates like *Bemisia*. Major facultative endosymbionts *Arsenophonus*, *Wolbachia, Hamiltonella, Cardinium,* and *Rickettsia* were evaluated for all whitefly genetic groupings and the infection frequencies were significantly correlated with the whitefly genotype. The current study showed that cassava whiteflies are mostly infected by single endosymbionts rather than multiple infections, as had been observed from previous studies elsewhere in Africa [39]. However, MED, which occurred on crop plants other than cassava, showed a higher level of endosymbiont coinfection. In this study MED had diverse secondary endosymbiont communities comprising *Arsenophonus*, *Rickettsia*, and *Wolbachia*. Similar studies have reported the presence of these endosymbionts in this mitotype [4,11,79,80,81,82]. MED individuals analyzed in this study were predominantly infected with *Arsenophonus*, which is comparable to findings from West Africa reporting a high prevalence of this endosymbiont in the ASL (=MED) mitotype [4]. By contrast, MED individuals collected from vegetables in Senegal were predominantly infected with *Hamiltonella* [11].

The cassava *B. tabaci* cryptic species in this study had diverse endosymbiont infections comprising *Arsenophonus, Rickettsia*, and *Wolbachia* as reported in other studies [39,79,83]. The work of Ghosh et al. [39] showed that cassava whiteflies are infected by *Arsenophonus, Rickettia, Wolbachia*, and *Cardinium* with the predominance of *Wolbachia* in Tanzania, Malawi, Uganda, and Nigeria. Tajebe et al. [79] reported *Arsenophonus*, *Rickettsia*, *Wolbachia*, *Hamiltonella*, and *Cardinium*, with *Arsenophonus* being the most prevalent in cassava *B. tabaci* whiteflies in Tanzania.

*Trialeurodes vaporariorum* was only infected with *Arsenophonus,* as noted elsewhere [84]. *Hamiltonella* and *Cardinium* were not detected in this study, although they have been reported in other studies on whiteflies collected from Africa [4,39,79,83]. Secondary endosymbionts in *B. tabaci* have been shown to have an influence on whitefly biology, survival, fecundity, heat tolerance, resistance/susceptibility to insecticides, and virus transmission [85]. Tajebe et al. [79] noted that the most striking feature of *B. tabaci* individuals sampled from the cassava virus pandemic that affected parts of Tanzania was the virtual absence of *Arsenophonus*. Furthermore, Ghosh et al. [46] reported that cassava *B. tabaci*, infected by *Arsenophonus* and *Rickettsia*, had decreased fitness and virus retention compared to whiteflies of the same type that were not infected by either endosymbiont. In our study, there was, overall, a much greater frequency of *Arsenophonus* in non-cassava whiteflies and *B. afer* compared to those *B. tabaci* (SSA4 and SSA1-SG2) individuals sampled from cassava. However, larger sample numbers of cassava *B. tabaci* would be required to draw stronger conclusions about possible differences in *Arsenophonus* frequency amongst the cassava-colonizing *B. tabaci* genotypes present in Cameroon. Since the absence of endosymbionts has been linked to greater fitness and virus retention capabilities, however, it will be important to monitor endosymbiont occurrence in future studies. For these reasons, knowledge about prevailing endosymbionts in surveyed whitefly populations is critical for influencing future research on the role of these bacteria in whiteflies and their effect on virus epidemics.

## 5. Conclusions

This work demonstrated the presence of six mitotypes of *Bemisia tabaci*, and two distinct clades of *Bemisia afer* and *Trialeurodes vaporariorum* on vegetables and cassava in Cameroon. *Bemisia tabaci* mitotypes identified included MED on vegetables and SSA1-SG1, SSA1-SG2, SSA1-SG5, SSA3, and SSA4 on cassava. The MED mitotype was widely distributed in all sampling regions and is almost certainly the main phytovirus vector in Cameroonian vegetable cropping systems. For the first time, we found the haplogroup SSA-ECA on cassava in Cameroon. This is a concerning new development, as this haplogroup is predominant in regions currently affected by the severe cassava mosaic virus disease (CMD) and cassava brown streak virus disease (CBSD) pandemics in Eastern and Central Africa. The whiteflies in this study were found to be infected with endosymbionts from three different genera (*Arsenophonus, Wolbachia*, and *Rickettsia*). None of the insects were infected by *Hamiltonella* and *Cardinium*. Moreover, the *Rickettsia* species, which are implicated in the resistance of insects to insecticides, entomopathogens, and natural enemies, was recorded in all whitefly species at varying levels. MED mitotype whiteflies were predominantly infected with *Arsenophonus*, which is implicated in the adaptability of whiteflies. These findings add to the knowledge of the diversity of whiteflies and associated endosymbionts, which, when combined, can influence virus epidemics and responses to whitefly control measures especially insecticides.

## Figures and Tables

**Table 1:** The range of climate characteristics (annual rainfall, relative humidity, and temperature) of the three agroecological zones covered in the study.

Region	Average Mean Temperature (°C)	Annual Relative Humidity (%)	Average Rainfall (mm)	Agroecological Zone
Southwest	22.6–33.6	66.1–86.2	2000–3700	Monomodal rainforest
Littoral	22.6–33.6	66.1–86.2	2000–3700	Monomodal rainforest (humid forest)
West	22.6–33.1	76.4–84.5	1800–2100	Western highland
Center	17.3–31.6	60–90	1500–2000	Bimodal rainforest (humid forest)

**Table 2:** Primer sequences and annealing temperatures used for PCR amplification of endosymbionts.

Target Gene	Primer Name	Sequence (5′→3′)	Reference	Amplicon Length	Annealing Temperature
*Portiera* 16S rDNA	28F 1098R	TGCAAGTCGAGCGGCATCAT AAAGTTCCCGCCTTATGCGT	[25]	1050 bp	58 °C
*Arsenophonus* 23S rDNA	Ars23S-1 Ars23S-2	CGTTTGATGAATTCATAGTCAAA GGTCCTCCAGTTAGTGTTACCCAAC	[30]	750 bp	58 °C
*Rickettsia* 16S rDNA	Rb-F Rb-R	GCTCAGAACGAACGCTATC GAAGGAAAGCATCTCTGC	[24]	960 bp	58 °C
*Wolbachia* *wsp* gene	81F 471R	TGGTCCAATAAGTGATGAAGAAACAAAAATTAAACGCTACTCCA	[39]	600 bp	53 °C
*Cardinium* 16S rDNA	Card-F Card-R	TAGACACACACGAAAGTTCATGT GCATGCAATCTACTTTACACTGG	[39]	650 bp	57 °C
*Hamiltonella* 16S rDNA	Hb-F Hb-R	TGAGTAAAGTCTGGGAATCTGG AGTTCAAGACCGCAACCTC	[18]	730 bp	58 °C

**Table 3:** Whitefly abundance on different crops in three different agroecological zones of Cameroon.

Region	Division	Agroecological Zone	Number of Fields	Crop (Number of Fields)	Whitefly Abundance (Mean)
Southwest	Dian Koupemanengouba	Monomodal rainforest	2	Cassava	12.5
Littoral	Moungo	Monomodal rainforest (humid forest)	3	Cassava	2.9
West	Nde	Western highland	5	Cassava (2) Tomato (3)	5.9 37.3
Center	Mefou et Afamba Mefou et Akono Mfoundi	Bimodal rainforest (humid forest)	14	Cassava (8) Tomato (1) Okra (3) Melon (1) Pepper (1)	10.7 22.0 31.6 60.0 18.0

**Table 4:** The six SNP-based haplogroups and associated mtCOI sequences mitotypes of cassava whitefly *Bemisia tabaci*.

KASP Haplogroup	Mitotypes (mtCOI)	References
SSA-ECA	SSA1-SG1 SSA1-SG2, SSA1-SG1/SG2, SSA4	[54,55,56,57]; in this study
SSA-ESA	SSA1-SG3, SSA1-SG2	[54,56,57,58];
SSA-WA	SSAA1-SG1, SSA1-SG5, SSA2, SSA4	[56,57]; in this study
SSA-CA	SSA1-SG1, SSA1-SG2	[56]
SSA2	SSA2, SSA3, SSA4	[54,56,57]
SSA4	SSA4, SSA1-SG2, SSA1-SG5	[56,57]; in this study

## Data Availability

GenBank accession number for nucleotide sequences https://www.ncbi.nlm.nih.gov/nuccore/?term=PP580858:PP580933[accn] accessed on 24 July 2024.
